# *Salmonella* Typhi meningitis in a 9-year old boy with urinary schistosomiasis: a case report

**DOI:** 10.1186/s13104-015-1030-2

**Published:** 2015-03-03

**Authors:** Flora Chacha, Stephen E Mshana, Mariam M Mirambo, Martha F Mushi, Rogatus Kabymera, Lisa Gerwing, Wilhelm Schneiderhan, Ortrud Zimmermann, Uwe Groß

**Affiliations:** Department of Pediatric and child Health Weill Bugando School of Medicine, Catholic University of Health and Allied Sciences, Mwanza, Tanzania; Department of Microbiology and Immunology Weill Bugando School of Medicine, CUHAS-Bugando, P.O. BOX 1464, Mwanza, Tanzania; Institute of Medical Microbiology, University Medical Center Goettingen, Goettingen, Germany

**Keywords:** Meningitis, *Salmonella* Typhi, Schistosoma haematobium

## Abstract

**Background:**

Cases of *Salmonella* Typhi meningitis have been rarely reported in infants. There are few documented cases of persistent salmonella bacteraemia in patients with schistosomiasis. A presented case highlights the importance of broadening the list of pathogens that can cause meningitis among older children in schistosomiasis endemic regions.

**Case presentation:**

The reported case is of a 9-year old sukuma-black African boy referred to Bugando Medical Centre with complaints of fever, abdominal pain, headache and generalized body weakness. On examination; the child was febrile (39°C) with neck stiffness and distended abdomen. Cerebrospinal fluid culture was positive for *Salmonella* Typhi. In addition on urine sediments microscopy, *Schistosoma haematobium* eggs were seen. The child improved clinically on ceftriaxone and praziquantel, and was discharged 3 weeks after admission.

**Conclusion:**

Complicated persistent salmonella infection should be considered in schistosomiasis endemic areas. More research should be done to confirm the association between salmonella infections and urinary schistosomiasis.

## Background

Salmonella infections are common causes of febrile illness in sub-Saharan Africa especially in areas where Human Immunodeficiency Virus (HIV) is endemic [1,2]. In addition about 29 · 1% of non-malaria bloodstream infections in Africa are due to *Salmonella enterica* with 58 · 4% of these due to non-typhoidal *Salmonella* [3]. In Malawi, non-typhoidal salmonella has been found to be the commonest cause of meningitis in neonates [4]. In infants and young adults, the usual infections due to salmonella include typhoid fever and gastroenteritis. However, in Africa bloodstream infections from nontyphoidal salmonella are common [4,5]. Worldwide, few cases of typhoid and non-typhoid salmonella meningitis have been reported in infants [4,6-8]. The reported cases have been associated with increased morbidity and mortality. Mortality rates as high as 40% have been reported in children with salmonella meningitis irrespective of the serotype [9-11]. We present a case of *Salmonella* Typhi meningitis in a 9-year old boy associated with peritonitis, neutropenia and urinary schistosomiasis.

## Case presentation

A 9-year old sukuma-black African boy was referred to the Bugando Medical Centre (BMC) from a district hospital with three weeks history of fever and abdominal distention and one week history of generalized body weakness and headache. This was the second admission in his life time; the first admission was 4 years ago due to severe malaria. He is 8^th^ child in the family of 10 children and he was fully immunized as per Tanzanian Expanded programme of immunization. There was no history of convulsion, loss of consciousness, vomiting and diarrhea. It was reported that the abdomen was grossly distended and tender. However, he was passing stool normally. Also, it was reported that he used amoxicillin and gentamicin at the district hospital with no improvement. On examination, he was febrile (39°C), ill looking, pale, and jaundiced. Abdomen was grossly distended, tender with shifting dullness. Liver and spleen were not palpably enlarged and there was no rebound tenderness. He had neck stiffness with negative Kernig’s signs. Lungs were clear on auscultation, first (S1) and second (S2) heart sounds were heard with a gallop rhythm. Respiratory rate was 46/minute while the heart rate was 120 beats/minute with oxygen saturation of 94% on ambient air. Provisional diagnosis of severe malaria was reached with differentials of meningitis, peritonitis and typhoid fever.

Lumbar puncture (LP) was done which revealed a xanthochromic cerebrospinal fluid(CSF) with total white blood cells (WBC) count of 52/cmm3 of which 88% were lymphocytes. A reactive mononuclear cell was 10% while pandy’s test was positive and cryptococcal antigen test was negative. On CSF gram stain, no organisms were seen. A nasogastric tube was inserted and the child was made nil by mouth due to distended abdomen. Intravenous ceftriaxone 1 g 24 hrly for 2 weeks and intramuscular artemether 64 mg stat then 32 mg daily for 7 days were initiated.

**Figure 1:**
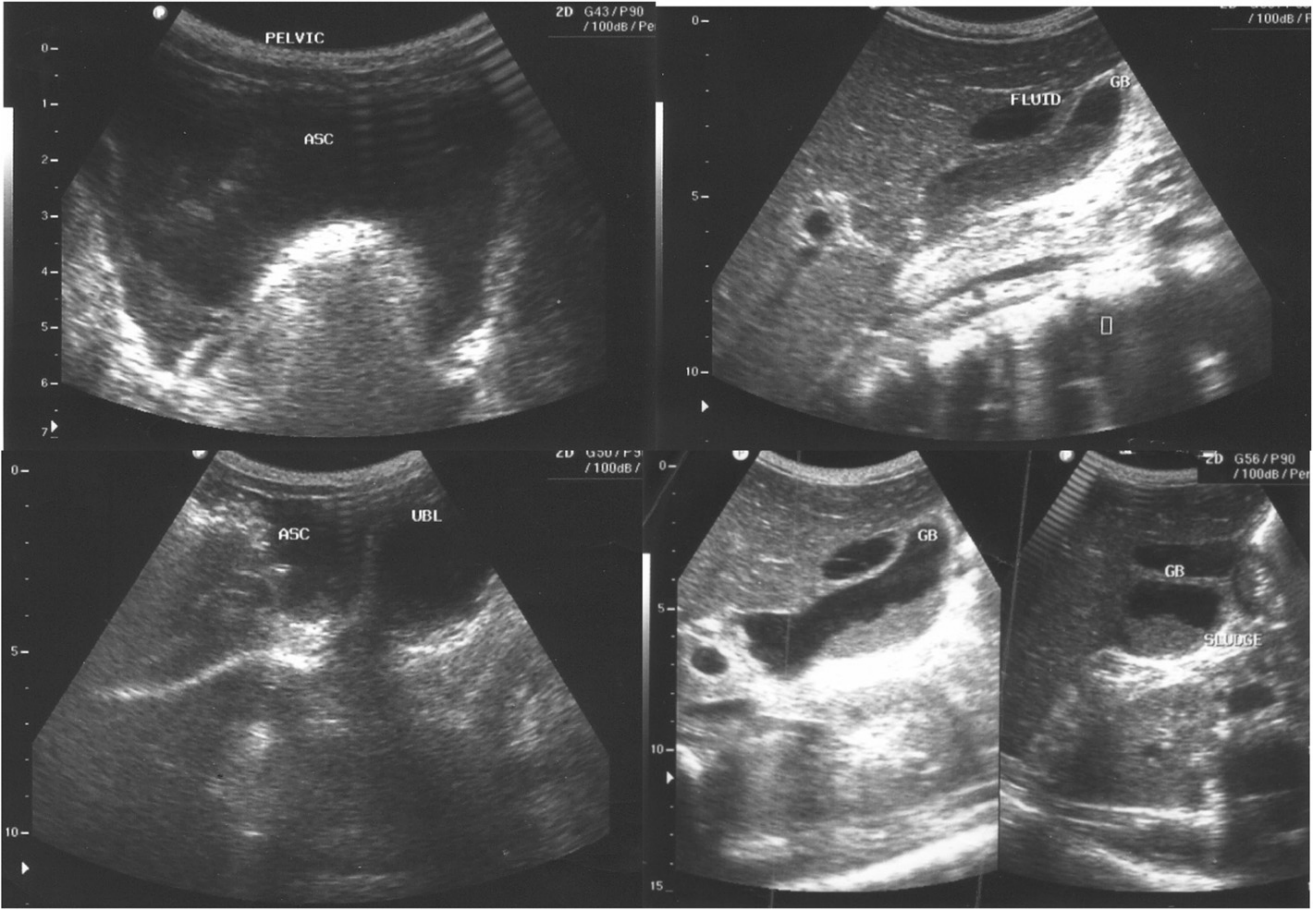
**Ultra sound showing accumulation of fluid in the peritoneal cavity and dilated thickened wall gall bladder: features consistence with peritonitis and cholecystitis.**

Abdominal ultrasound revealed increased free fluid in the peritoneal cavity, slightly enlarged liver and distended gall bladder with thickened wall (Figure 1). In other laboratory investigations; hemoglobin level of 8.4 g/dl, total WBC count of 3,100/mm3 (neutrophils 75%, lymphocyte 19%) and platelet count of 181,000/μl were detected.

Widal test results against O (*Salmonella* Typhi O) and H (*Salmonella* Typhi H) were both > 1:160. The rapid Hepatitis B surface antigen, HIV rapid test and blood slide for malaria parasites were all negative. Slightly elevated liver enzymes were noted; alanine transaminase (51.63 U/L) and aspartate transaminase (54 U/L). Bilirubin levels were found to be high with total bilirubin of 176.3 μmol/L and direct bilirubin of 133.5 μmol/L.

Urine microscopy was positive for *Schistosoma haematobium* ova and red blood cells (RBC) (10-20/high power field). On stool microscopy no ova/cysts were seen but numerous white blood cells were seen. CSF culture on blood agar plate (Oxoid Limited, UK) was positive for a significant growth of pure culture of gram negative short rods bacteria. During subsequent culture, growth was also seen on MacKonkey agar (Oxoid Limited, UK). In-house biochemical identification tests provided inconclusive results. Further identification using MALDI-TOF Mass Spectrometry (Bruker, Germany) and Kauffmann White agglutination identified the isolate as *Salmonella* Typhi which was typed to be lysotypeE1a. Susceptibility testing using VITEK-2 showed the isolate to be resistant to ampicillin, ampicillin/sulbactam, tobramycin and co-trimoxazole while being sensitive to piperacillin/tazobactam, cefotaxime, ceftriaxone, ceftazidime, cefpodoxime, imipenem, meropenem, ertapenem, ciprofloxacin (minimum inhibitory concentration (MIC) = 0.047 mg/L), and moxifloxacin.

The condition of the patient improved clinically on ceftriaxone, paracetamol and praziquantel 400 mg. Despite the patient being weak, he was discharged following relative’s request on day 21 to be followed at pediatric outpatient clinics.

## Discussion

*Salmonella* Typhi sepsis with meninges involvement is an uncommon complication in adults and older children [1,12]. Few cases have been reported in infants and have been associated with increased morbidity and mortality [4,6-10]. In the present case, a 9-year old boy was confirmed to have *Salmonella* Typhi meningitis based on CSF culture. Although this case presented at the beginning with symptoms suggestive of typhoid fever; the diagnosis of typhoid fever was not reached due non-specific presentation of typhoid fever in endemic area; signifying the need for improved access to diagnostic microbiology in Africa [13]. Usually, typhoid fever presents with continual fever, accompanied by increasing fatigue, abdominal distension, constipation, abdominal pain, frontal headaches, malaise, anorexia, nausea and vomiting [14,15]. Later in the disease course the patient may present with jaundice due to elevated conjugated bilirubin as a result of endotoxin induced cholestasis [16]. All these features appeared in the present case at the district hospital. However, due to limited diagnostic tests and lack of high index of suspicion, lumber puncture and blood culture were not done. Though positive in our patient, the Widal test is of limited value in a hyperendemic country such as Tanzania since a large number of patients present with a positive Widal reaction due to previous infections.

Acute bacterial meningitis due to *Salmonella* Typhi is uncommon in adults and older children; however, when suspected, appropriate treatment should be initiated as early as possible to prevent associated morbidity and mortality. The diagnosis of typhoid fever at the district hospitals is a challenge in Tanzania due lack of diagnostic facilities. In addition the treatment is also a challenge because commonly available antibiotic for sepsis are ampicillin/amoxicillin and gentamicin of which most isolates causing sepsis are resistant. Moreover, gentamicin is usually not effective for facultative intracellular organisms such as salmonellae [17-19].

Although not proven, we cannot exclude a promoting effect on typhoid fever complicating meningitis by co-infection with *Schistosoma haematobium* in this child. A positive association between schistosomiasis and *Salmonella* Typhi has been described in patients as well as in experimental mouse models [20-23]; this could be explained by the argument that the parasite facilitates invasion via the urinary tract, so meningitis would follow bacteremia. Nevertheless; to date no case has been published associating urinary schistosomiasis and *Salmonella* Typhi meningitis; more research on this interaction is needed especially in endemic areas to confirm the argument.

Based on this case and other cases in infants and neonates, salmonella meningitis should be considered whenever gram-negative bacteria are seen in CSF in patients living in sub-Saharan Africa. Clinicians should have high index of suspicion especially if patients present with signs and symptoms of typhoid fever. The appropriate treatment for salmonella meningitis is a combination of ciprofloxacin (especially in adults) and ceftriaxone/cefotaxime [19]. Although chloramphenicol penetrates well through meninges, currently high resistance rates against this antibiotic to *Salmonella* Typhi argue against its use in sub-Saharan Africa [24]. In order to prevent relapse, high dose of a third-generation cephalosporin for at least 4 weeks is recommended [19,25]. In this case, history of low grade fever associated with abdominal distension which was followed by jaundice indicates that the disease started as typhoid fever with dissemination to the meninges. Lack of blood culture is the limitation to confirm this probable course.

## Conclusion

We report this rare case to alert clinicians that *Salmonella* Typhi could contribute significantly as a possible etiology of bacterial meningitis in children in developing countries. The diagnosis requires proper investigation with high index of suspicion so that appropriate management can be instituted timely. Lastly, establishing bacteriological laboratories would largely contribute to rapidly differentiate between malaria and other bacteriological causes of diseases in developing countries.

## Consent

Written informed consent was obtained from the patient’s parents for publication of this Case Report and any accompanying images. A copy of the written consent is available for review by the Editor-in-Chief of this journal.

## Abbreviations

ASC: Ascites
CSF: Cerebrospinal fluid
GB: Gall bladder
HIV: Human immunodeficiency virus
MIC: Minimum inhibitory concentration
TOF: Time-of-flight
UBL: Urinary bladder
UK: United Kingdom
WBC: White blood cells
